# Electrical injuries. Biological values measurements as a prediction factor of local evolution in electrocutions lesions

**Published:** 2014-06-25

**Authors:** R Teodoreanu, SA Popescu, I Lascar

**Affiliations:** “Carol Davila” University of Medicine and Pharmacy, Bucharest, Romania Plastic Surgery and Reconstructive Microsurgery Department, Clinical Emergency Hospital, Bucharest, Romania

**Keywords:** electrical injuries, electrocutions, electrically-injured patient, CK level, carbonization

## Abstract

Abstract

Rationale. Taking into account the incidence and the severity of electrocutions, we consider it extremely necessary to find effective, appropriate and particularized therapeutic solutions aimed at improving the survival, decreasing the mortality, ensuring a superior functional and aesthetic effect and facilitating the social reintegration.

Given the severity of the general condition of the electrically injured patient and the fact that any worsening of the lesions has a systemic echo, the selection of the timing for re-excision is very important.

The postponement of the surgical timing can break the precarious metabolic equilibrium and can hasten the installation of the multisystem organ failure (MSOF).

Objective. The study is intended to establish a possible connection between the clinical evolution of the electrically injured patient and the dynamics of three important biological parameters, able to provide data concerning the therapeutic attitude to be followed.

The patients with a diagnostic of high-voltage electrocution, who will be admitted to the Clinic, will be followed for a period of 2 years. The parameters to be followed daily will be:

- Creatin-kinase, as a marker of muscular damage

- Hemoglobin, as a marker of tissue oxygenation

- Leukocytes, as an indicator of a possible septic evolution.

The therapeutic alternatives, including the administration of antiplatelet drugs will be studied.

Methods and Results. In the period October 2010-June 2013 a total of 12 cases of high-voltage electrocution were admitted in our clinic. Among these, some could be placed in the study of 7 cases, as the remaining patients died within the first 24 hours of hospitalization due to the endured lesions.

All the patients were admitted to the ICU ward that supported the treatment and monitoring until their stabilization, at which time they were transferred to the ward.

All the patients received anti-thromboxane treatment from their admission (injectable NSAIDs associated with antisecretory drugs). By mutual agreement with ICU service, Dipyridamole was not introduced because of the “steal effect” in the viable areas to the detriment of the already ischemic areas, the drug effect being obvious in vitro, but hard to be proven in the clinical case.

The relationship between the CK level and the clinical appearance of the ischemic areas is relative. We cannot conclude that an increased level of CK is equivalent to an enlarged ischemic area and even less it does not provide us direct information concerning the best time for re-excision.

The presence of a viable blood supply around the necrotic tissue will lead to an important resorption of degradation products in that area, a quasinormal level of CK having no value. The sealing of the necrosis areas and the lack of immediate resorption does not have a positive prognostic value. Taking into account that the electrocutions are mostly multiple injuries, the CK level can increase even after some muscular damages, fractures, independent of the actual electrocution lesion.

In one case, the patient suffered from electrocution at both thoracic limbs. With the carbonization of the hands and grifa installed up to the level of the elbow fold, he stayed for 6 hours at the accident site until he had been recovered. At the moment of presentation to the hospital, his consciousness condition was satisfactory but the CK level was of over 20000 IU, becoming rapidly non-detectable, in combination with black urine. The patient's condition deteriorated quickly, and, although the bilateral shoulder disarticulation has been carried out, he died in the next 12 hours.

Discussion. As a conclusion, the CK level did not prove itself a prognostic for the surgical timing or the actual surgical attitude and could be influenced by a whole series of factors, dependent or not on the electrocution lesion.

A radical attitude is to be preferred in cases with established ischemia; the prognostic being the more reserved the larger the damage and the longer the period of time from the event. The established treatment is of renal support and treatment of acute renal injury (AKI) subsequently installed.

An increased level of leukocytes is always present as in any severe trauma, even if there are no immediate signs of infection of the electrocution lesions.

Taking into account that the electrocution lesion as well as the one caused by burning destroys the natural defense barrier represented by the skin, the infection risk is major and that is why the therapeutic protocol stipulates the immediate establishment of a treatment with broad-spectrum antibiotics or with an association of antibiotics.

The increase of the leukocytes level under antibiotics treatment involves either the contamination with a germ that is not sensitive to the respective antibiotic or the persistence of necrosis areas which secondarily infect, and where antibiotic penetration is very low. Therefore, the excision of the compromised tissues is an absolute necessity.

In terms of prognostic, the increase of the leukocytes number signified an insufficient excision and indicated the resuming and deepening of the excisions. Taking into account that the patient has been admitted through the ICU service, the risk of contracting severe infections with selected germs is real. Another risk is that of infection with Clostridium difficile following the prolonged utilization of broad-spectrum antibiotics, especially in patients with associated diseases and reduced immunity per primam.

The existence of completely separate circuits should solve the problem of contamination with bacteria of selected species; unfortunately, in our cases, we have faced this problem and the utilization of last choice antibiotics (Imipenem, Vancomycin, Targocid, etc.) as well as the association of immunoglobulins was necessary.

All the patients admitted in the study received anti-thromboxane drugs in order to limit the ischemic process at tissue level. Despite the efforts we have made, the lack of blood and its derivatives or simply the negligence in patient monitoring, allowed the decrease, even transient of the Hb level, sometimes only for a few hours, but enough to allow the deepening of the ischemic lesions.

Excisions were carried out in all the patients in emergency or even amputations of the extremities, with the wish to limit the extension of the ischemic lesions and the resorption of cell degradation products.

The amputations performed in emergency did not always represent a saving solution; however, they remained the most effective measures when they were carried out immediately after the accident and obviously in viable tissue.

The increase of CK is not an indicative factor itself in making re-excisions but orients the therapeutic approach, the utilization of the dialysis when the values do not decrease by treatment for renal support and the forcing of diuresis is required.

The normalization of CK indicates the time when we can start the covering of the defects resulted as a consequence of the excisions.

The level of the leukocytes represents both a prognostic factor and an indicative factor for the re-excision of the ischemic areas. An increased level under antibiotic therapy signifies either an incomplete excision or the contamination with flora resisting to the antibiotic that has been used.

In the light of findings in the caring of the patients with electrocutions, I propose several caring/assessment protocols for the severe electrically injured patient.

## Perspective

Injuries from artificial electricity have been reported for almost 300 years. The first death recorded caused by the electrical current from an artificial source was reported in 1879, when a carpenter in Lyons, France, inadvertently contacted a 250-volt AC generator [1]. The first U.S. fatality occurred in 1881, when a local inebriate, Samuel W. Smith, passed out on a similar generator in front of a crowd in Buffalo, New York. The apparent painlessness of his death impressed the crowd, and electrocution was considered a “humane” way of execution. In 1890, William Kemmeler was the first man to be put to death in New York State’s electric chair [2].

 Electrical burns account for 4% to 6.5% of all admissions to burn units in the United States and for approximately 1000 fatalities per year in the United States [3]. Most electrical fatalities and adult admissions to burn centers from electrical injury are occupationally related. Children have a predisposition to injuries from low-voltage sources, such as electric cords, because of their limited mobility within a relatively confined environment [4]. During adolescence, however, a more active exploration of the environment is possible and may lead to more severe high-voltage injuries or death.

 At the time of presentation, documentation of injuries is important not only for the immediate resuscitation of the victim but also medico-legally. Nearly all cases of electrical injuries eventually involve litigation for negligence, product liability, or worker compensation.

**Principles of disease**

**Physics of Injury**

 The exact pathophysiology of the electrical injury is not well understood because of the large number of variables that cannot be measured or controlled when an electrical current passes through tissues. With high-voltage injuries, most of the injury appears to be thermal and most histological studies reveal coagulation necrosis consistent with thermal injury [5,6]. Lee et al [7] proposed the theory of electroporation in which electrical charges too small to produce thermal damage, cause protein configuration changes that threaten cell wall integrity and cellular function. The nature and severity of electrical burn injuries are directly proportional to the current strength, resistance and duration of current flow.

**Mechanisms of Injury**

 The primary electrical injury is represented by burns. Secondary blunt trauma results from falls or being thrown from the electrical source by an intense contraction of muscles. Electrical burns can be classified into four different types [9].

 Types of Electrical Burns

 Direct contact

 Electrothermal heating

**Indirect contact**

 Flame

 Flash

 Heating of tissues secondary to current causes electro-thermal burns. Usually, these burns are a result of a low voltage shock with a limited affected area. Severe electro-thermal burns can occur, however, if a person grips a high voltage conductor. The prolonged flow of current can result in significant burns anywhere along the current path. Typically, the skin lesions of electro-thermal burns are well-demarcated, deep partial to full-thickness burns.

 The most destructive indirect injury occurs when a victim becomes part of an electrical arc. An electrical arc is a current spark formed between two objects of different potential that are not in contact with each other, usually a highly charged source and the ground. Because the temperature of an electrical arc is of approximately 2500°C, it causes very deep thermal burns at the point where it contacts the skin [8]. In arcing circumstances, burns may be caused by the heat of the arc itself, electro-thermal heating due to current flow, or by flames that result from the ignition of clothing. Instead of jumping in the form of a discrete arc causing contact point burns, current may jump the gap by splashing across the entire body. These splash burns may cover a large portion of the body but are generally only of partial thickness [9].

 At the time of presentation, it is often difficult to determine the mechanism of injury that caused an electrically injured patient’s burns. Electro-thermal heating is the main cause of muscle damage and it is almost exclusively seen in high voltage accidents with prolonged (seconds) contact and current flow [9].

 The histological change seen in the muscle injury that results from the direct contact with an electrical source is coagulation necrosis with the shortening of the sarcomere [6,8]. Muscle damage can be spotty, so areas of viable and nonviable muscle are often found in the same muscle group. Periosteal muscle damage may occur even though overlying muscle appears to be normal. Similarly to the muscle damage, serious vascular damage usually occurs only after a high voltage accident.

 Vascular damage is greatest in the media. This can lead to delayed hemorrhage when the vessel eventually ruptures [2,8,10]. Intimal damage may result in either immediate or delayed thrombosis or vascular occlusion as edema and clots form on the damaged internal surface of the vessel over a period of days [10]. The injury is usually the most severe in the small muscle branches, where the blood flow is slower [11]. This damage to small muscle arteries, combined with mixed muscle viability that is not visible to gross inspection, creates the illusion of “progressive” tissue necrosis.

 Immediate arterial thrombosis will result in the absence of a pulse on an initial examination. The absence of a pulse, however, can also be due to transient vascular spasm. Pulselessness resulting from vascular spasm should resolve within a few hours. If pulselessness persists after this time, serious vascular injury is likely to occur.

 Damage to neural tissue may also occur via several mechanisms. A nerve may demonstrate an immediate drop in conductivity as it undergoes coagulation necrosis similar to that observed in the muscle. In addition, it may suffer indirect damage as its vascular supply or myelin sheath is injured or as progressive edema results in a compartment syndrome. The signs of neural damage may develop immediately or may be delayed from hours to days. Since the skull is a common contact point, the brain is commonly injured. Histologic studies of the brain reveal focal petechial hemorrhages in the brain stem, cerebral edema, and widespread chromatolysis (the disintegration of chromophil bodies of neurons) [8].

 Exposure to electricity may cause an immediate death from asystole, ventricular fibrillation, or respiratory paralysis, depending on the voltage and pathway.

**Head and Neck**

 The head is a common point of contact for high-voltage injuries, and the patient may exhibit burns as well as neurological damage. Cataracts develop in approximately 6% of the cases of high-voltage injuries, especially whenever electrical injury occurs in the vicinity of the head [17]. Although cataracts may be present initially or develop shortly after the accident, they more typically appear months after the injury. Visual acuity and fundoscopic examination should be performed at presentation. Referral to an ophthalmologist familiar with electrical cataract formation may be necessary.

**Cardiovascular System**

 Cardiac arrest, from either asystole or ventricular fibrillation, is a common condition present in electrical accidents. Other electrocardiographic (ECG) findings include sinus tachycardia, transient ST segment elevation, reversible QT segment prolongation, premature ventricular contractions, atrial fibrillation, and bundle branch block [2,19]. Acute myocardial infarction is reported but is relatively rare [20,21]. Damage to skeletal muscles may produce a rise in the CPK-MB fraction, leading to a spurious diagnosis of myocardial infarction in some settings [21,22].

** Skin**

 Other than cardiac arrest, the most devastating injuries that accompany an electrical injury are burns, which are most severe at the source and ground contact points. The most common sites of contact with the source include the hands and the skull. The most common areas of ground are the heels. A patient may have multiple source and ground contact points. Burns in severe electrical accidents often appear as painless, depressed, yellow-gray, punctate areas with central necrosis, or the areas may be mummified [14]. High-voltage current often flows internally and can create massive muscle damage. If contact was brief, however, minimal flow may have occurred and the visible skin damage may represent nearly all of the damage. One should not attempt to predict the amount of underlying tissue damage from the amount of cutaneous involvement.

 A peculiar type of burn associated with electrical injury is the “kissing burn”, which occurs at the flexor creases [8]. As the current causes flexion of the extremity, the skin of the flexor surfaces at the joints touches. Combined with the moist environment that often occurs at the flexor areas, the electric current may arc across the flexor crease, causing arc burns on both flexor surfaces and extensive underlying tissue damage.

 Electrical flash burns are usually superficial partial-thickness burns, similar to other flash burns. Isolated thermal burns may also be seen when clothing ignites.

 The total body surface area affected by burns in electrical injury averages 10% to 25%. Severe burns to the skull, and occasionally to the dura, are reported [25,26]. The most common electrical injury seen in children less than 4 years of age is the mouth burn that occurs from sucking on a household electrical extension cord [4].

 These burns usually represent local arc burns, may involve the orbicularis oris muscle, and are especially worrisome when the commissure is involved because of the likelihood of cosmetic deformity. A significant risk of delayed bleeding from the labial artery exists when the eschar separates. Damage to developing dentition is reported, and referral to an oral surgeon familiar with electrical injuries is recommended [27].

**Extremities**

 In high-voltage injuries, muscle necrosis can extend to sites distant from the observed skin injury, and compartment syndromes occur as a result of vascular ischemia and muscle edema. Decompression fasciotomy or amputation is often necessary because of extensive tissue damage [28,29]. Massive release of myoglobin from the damaged muscle may lead to myoglobinuric renal failure. Vascular damage from the electrical energy may become evident at any time [10,11]. Pulses and capillary refill should be assessed and documented in all extremities, and neurovascular checks should be repeated frequently. Because the arteries are a high-flow system, heat may be dissipated fairly well and cause little apparent initial damage but result in subsequent deterioration. The veins, on the other hand, are a low-flow system, allowing the heat energy to cause a more rapid heating of the blood, resulting into thrombosis. Consequently, an extremity may initially appear edematous. With severe injuries, the entire extremity may appear mummified when all tissue elements, including the arteries, suffer from coagulation necrosis.

 Damage to the vessel wall at the time of the injury may also result in delayed thrombosis and hemorrhage, especially in the small arteries to the muscle [10,11].

 This ongoing vascular damage can cause a partial-thickness burn to develop into a full-thickness burn as the vascular supply to the area diminishes. Progressive loss of muscle because of vascular ischemia downstream from damaged vessels may mandate repeated deep debridements [11].

**Skeletal System**

 Fractures of most of the long bones caused by the trauma associated with electrical injury are reported [31,32]. Both posterior and anterior shoulder dislocations caused by tetanic spasm of the rotator cuff muscles are also reported, as well as spinal fractures. As far as the electrical injury is concerned, numerous types of fractures and dislocations are reported with lightning injury [5].

**Nervous System**

 In high-voltage injuries, loss of consciousness may occur but is usually transient unless there is a significant concomitant head injury. Prolonged coma with eventual recovery is also reported. Patients may exhibit confusion, flat affect, and difficulty with short-term memory and concentration [12-16,30].

 Electrical injury to the central nervous system (CNS) may cause a seizure, either as an isolated event or as part of a new-onset seizure disorder [30]. The other possible causes of seizures, such as hypoxia and traumatic CNS injury, should be considered. Neurologic symptoms may improve, but long-term disability is common. Lower extremity weakness is commonly undiagnosed until ambulation is attempted [31].

 In high-voltage exposures, spinal cord injury may result from fractures or ligamentous disruption of the cervical, thoracic, or lumbar spine [31-33].

 Neurological damage in patients without evidence of spinal injury seems to follow two patterns, immediate and delayed. Patients with immediate damage have symptoms of weakness and paresthesias developing within hours of the insult [30].

 Lower extremity findings are more common than upper extremity findings. These patients have a good prognosis for partial or complete recovery. Delayed neurological damage may present from days to years after the insult. The findings usually fall into three clinical pictures: ascending paralysis, amyotrophic lateral sclerosis, or transverse myelitis [33]. Motor findings predominate. Sensory findings are also common, but they may be patchy and may not match the motor levels. Although recovery is reported, the prognosis is usually poor [31].

**Other Viscera**

 Injury to the lungs may occur because of associated blunt trauma but is rarer than the electrical current, perhaps because air is a poor conductor. Injury to solid visceral organs is also rare, but damage to the pancreas and liver is reported [35]. Injuries to hollow viscera, including the small intestine, large intestine, bladder, and gallbladder, are also reported [35,36].

**Other Low-Voltage Injuries**

 An accurate history is essential to ensure that an apparent low-voltage injury was not caused by the discharge from a capacitor (as in the repair of a television, microwave oven, or computer monitor) or other high-voltage source. Although burns from low-voltage sources are usually less severe than those from high-voltage sources, patients may still complain of paresthesias for an extended period, experience cardiac dysrhythmias, or have cataracts develop if the shock occurs close to the face or head [4].

**Complications**

 Cardiac arrest generally occurs only at the initial presentation or as a final event after a long and complicated hospital course. Many complications are similar to those of thermal burns and crush injuries, including infection, clostridial myositis, and myoglobinuria. The incidence of acute myoglobinuric renal failure has decreased since the widespread adoption of aggressive alkalinized fluid resuscitation. Fasciotomies or carpal tunnel release may be necessary for the treatment of compartment syndromes [28,29]. Tissue loss and major amputations are common with severe high-voltage injuries and result in the need for extensive rehabilitation.

 Neurologic complications such as the loss of consciousness, peripheral nerve damage, and delayed spinal cord syndromes may occur [16,30-33,34]. Damage to the brain may result in a permanent seizure disorder [18]. Long-term neuropsychiatric complications include depression, anxiety, and inability to continue in the same profession, aggressive behavior, and suicide [37]. Stress ulcers are the most common gastrointestinal complications after burn ileus. Abdominal injuries from ischemia, vascular damage, burns, or associated blunt trauma may be overlooked initially [14,35,36]. The most common causes of hospital mortality are pneumonia, sepsis, and multisystem organ failure [24].

** Differential considerations**

 Electrical injuries are usually self-evident from historical surroundings, except in the case of bathtub accidents, instances when no burns occur, or foul play. It is important to determine the mechanism of burn injury because flash burns have a much better prognosis than arc or conductive burns. Alterations in consciousness or seizures can be caused by the electrical injury or result from an associated traumatic brain injury.

** Management**

 Prehospital

 Securing the Scene

 When first reaching the scene, prehospital personnel should secure the area so that bystanders and rescuers do not sustain other injuries. For high-voltage incidents, the power source must be turned off. Although many approaches to achieving this goal are recommended, the safest approach is to involve the local power company in high-voltage accidents. Accidents involving discrete electrical sources that are easily disconnected through a circuit box or switch are easier to manage, although rescuers should still ensure that the power is off before approaching the victim. The use of electrical gloves by emergency medical service (EMS) personnel is very dangerous. A microscopic hole in a glove can result in an explosive injury to the hand inside it, as thousands of volts from the circuit concentrate there to enter the glove.

 Initial Resuscitation

 Electrical injury victims may require a combination of cardiac and trauma care because they often suffer blunt injuries and burns as well as possible cardiac damage. Spinal immobilization is indicated whenever associated spinal trauma is suspected. Fractures and dislocations should be splinted, and burns should be covered with clean, dry dressings.

 All the patients with conductive injury should have at least one large-bore intravenous (IV) line established. An electrical injury should be treated like a crush injury, rather than a thermal burn, because of the large amount of tissue damage that is often present under normal-appearing skin. As a result, none of the formulas for IV fluids based on percentage of burnt body surface area is reliable. Hypotensive patients should initially receive a bolus of 20 ml/kg of isotonic fluid, and subsequent fluid management then based on the patient’s vital signs and clinical status.

 ED

 Assessment

 The victim of an electrical injury may not be able to give an adequate history, either because of the severity of injury and accompanying shock and hypoxia or because of unconsciousness or confusion that often accompanies less severe injuries. The history obtained from bystanders and the paramedics regarding the type of electrical source, duration of contact, environmental factors at the scene, and resuscitative measures provided can be helpful.

 Resuscitative efforts should be continued in the ED with adequate fluid administration and insertion of a Foley catheter for patients with high-voltage injury and extensive burns. Fluids should be administered at a rate sufficient to maintain a urine output of at least 0.5 to 1.0 ml/kg/hr in the absence of heme pigment in the urine and 1.0 to 1.5 ml/kg/hr in its presence.

 Cardiac monitoring is indicated for severely injured patients and for those who have the indications listed in Box 7.76

 Indications for Electrocardiographic Monitoring

 Cardiac arrest

 Documented loss of consciousness

 Abnormal ECG

 Dysrhythmia observed in prehospital or ED setting

 History of cardiac disease

 Presence of significant risk factors for cardiac disease

 Concomitant injury severe enough to warrant admission

 Suspicion of conductive injury

 Hypoxia

 Chest pain

 All high-voltage injury victims and low-voltage victims with cardiorespiratory complaints should have an ECG and cardiac isoenzyme determinations. Although ECG changes and dysrhythmias are common with electrical injuries, anesthesia and surgical procedures performed in the first 48 hours of care can be accomplished without cardiac complications [38,39]. The patient’s clinical status should guide the use of invasive monitoring with central venous pressure catheters, intracranial pressure monitors, and Swan-Ganz catheters [5,40]. Victims of electrical injuries with altered mentation should have a computed tomography (CT) scan performed. 

 Ancillary Tests

 The laboratory evaluation of the patient sustaining an electrical injury depends on the extent of the injury. All the patients with evidence of conductive injury or significant surface burns should have the following laboratory tests: complete blood count (CBC), electrolyte levels, serum myoglobin, blood urea nitrogen (BUN), serum creatinine, and urinalysis. Patients with severe electrical injury or suspected intra-abdominal injury should also have obtained pancreatic and hepatic enzymes and coagulation profile [19]. The clinician should consider ordering a type and cross-match, particularly if major debridements are necessary. Arterial blood gas analysis is indicated if the victim needs ventilatory intervention or alkalinization therapy.

 All patients should be evaluated for myoglobinuria, a common complication of high voltage electrical injury. If the urine is pigmented or the dipstick examination of the urine is positive for blood, and no red blood cells (RBCs) are seen on microscopic analysis, assume the patient has myoglobinuria and treat accordingly.

 Creatine kinase (CK) levels should be drawn and isoenzyme analysis performed.

 Peak CK levels are shown to predict the amount of muscle injury, risk of amputation, and length of hospitalization; the clinical value of a single level in the acute setting, however, is not established. Cardiac enzyme levels should be interpreted with care when diagnosing myocardial infarction in the setting of electrical injury. The peak CK level is not indicative of myocardial damage in electrical injury because of the large amount of skeletal muscle injury. Skeletal muscle cells damaged by electrical current can contain as much as 20% to 25% CK-MB fraction, suggesting injured skeletal muscle rather than myocardial injury as a possible source of an elevated CK-MB fraction [22]. CK-MB fractions, ECG changes, thallium studies, angiography, and echocardiography correlate poorly in acute myocardial infarction following electrical injury. Other cardiac enzymes (such as troponin) are not well studied in electrical injury but may prove useful in determining myocardial injury.

 All patients sustaining an electrical injury should receive cardiac monitoring in the ED and an ECG despite the source voltage. 

 Radiographs of the cervical spine should be performed if spinal injury is clinically suspected. Radiographs of any other areas in which the patient complains of pain or has an apparent deformity should be performed. Angiography is not shown to be useful in the planning of debridements or amputations and is not routinely indicated [11]. Technetium pyrophosphate scanning to detect areas of clinically unsuspected myonecrosis can be useful [23,28]. Nonviable muscle will appear as cold spots on the scan, lacking the normal level of uptake. Hot spots on the scan may consist of 20% to 80% viable muscle and should be clinically followed [28,40,41]. CT or magnetic resonance imaging may be useful in the evaluation of associated trauma and are essential for evaluation of possible intracranial injuries, particularly if the Glasgow Coma Scale score does not progressively improve.

**Specific Therapies**

**Rhabdomyolysis**

 Patients with heme pigment in the urine should be assumed to have myoglobinuria until the diagnosis can be excluded by more specific testing. Alkalinization of the urine increases the solubility of myoglobin in the urine, increasing the rate of clearance. Urine output should be maintained at 1.0 to 1.5 ml/kg/hr until all traces of myoglobin have cleared from the urine while the blood is maintained at a pH of at least 7.45 using sodium bicarbonate. Furosemide or mannitol may be used to cause further diuresis. The recommended dose of mannitol is 25 g initially, followed by 12.5 g/kg/hr, titrated as needed to maintain urine flow greater than 50 ml/hr. Unlike with high-voltage injuries, rhabdomyolysis is rare with lightning injuries.

**Burn Wound Care**

 Cutaneous burns should be dressed with antibiotic dressings, such as sulfadiazine silver (Silvadene). Mafenide acetate (Sulfamylon) is occasionally used for selected burns; however, its use may result in electrolyte abnormalities because of its carbonic anhydrase inhibitory activity.

 Electrical burns are especially prone to tetanus, and patients should receive tetanus toxoid and tetanus immune globulin based on their immunization history. Clostridial myositis is common, but prophylactic administration of high-dose penicillin to prevent clostridial myonecrosis is controversial and should not be used without discussion with the managing surgeon or burn unit. In general, systemic antibiotics are not used unless culture or biopsy proves infection is present.

**Extremity Injuries**

 Current management of electrical injuries of the extremities favors early and aggressive surgical management, including early fasciotomy, carpal tunnel release, or even amputation of an obviously nonviable extremity [29,42]. Extremities should be splinted in a functional position to minimize edema and contracture formation. The hand should be splinted in 35- to 45-degree extension at the wrist, 80- to 90-degree flexion at the metacarpophalangeal joints, and almost full extension at the proximal and distal interphalangeal joints. This position minimizes edema formation. During the first several days of hospitalization, frequent monitoring of the neurovascular status of all extremities is essential [43].

**Summary of the paraclinical picture**

 - hemoconcentration 

 - anaemia 

 - metabolic acidosis 

 - massive hemoglobinuria 

 - increase of creatinine and creatine-phosphokinase 

 - myoglobinuria 

 - increase of AST and ALT, LDH, leukocytes 

**Importance of paraclinical picture monitoring **

 Depending on the dynamics of the biological constants, the unfavorable local evolution can be anticipated with the extension of necroses and the timing for re-excision can be chosen before the deterioration of the patient's general condition and occurrence of systemic complications. 

 It is associated with photographic documentation and histopathological examinations. 

 It allows the discussion of the therapeutic alternatives. 

**Pathophysiology **

 At the cell level: disturbances of the cell membrane function, with direct echo on the function of vital organs, especially of the heart and CNS. 

 At local level, the lesions are the consequence of Joule effect – when the electric current flows through the tissues, a part of the electric energy is converted into thermal energy, depending on their resistance. 

 The specific lesions represent the result of excessive heating of the tissues after the passage of the electric current through them. 

 The decrease of dermal ischemia following electrocutions can theoretically limit the necrosis in the coagulation area. It has been demonstrated that acetyl salicylic acid, methylprednisolone, indometacin, imidazole, dipyridamole and methimazole prevent the dermal ischemia, suggesting that prostaglandins and/or thromboxanes have a role in this pathogenesis. 

 The specific anti-prostaglandin antibodies (anti PGE2, PGF2 alpha, PGI2, TXA2) were put into contact with burnt guinea pig bioptic tissue, at various intervals post-burning.

 By immunoperoxidase technique, the presence of the specific metabolites of arachidonic acid was evidenced. 

 The coagulated tissue contained high levels of PGE2 and TXA2. 

 The effect of three thromboxane inhibitors (imidazole, methimazole and dipyridamole) on the dermal ischemia was studied. The studies were conducted with Xenon 133-labeled tissue. The half time of xenon was prolonged in the ischemic areas, but it was short in the ischemic areas treated with anti-thromboxanes. 

 Repeated studies with anti-thromboxane and anti-prostaglandin antibodies on animal tissues treated with thromboxane inhibitors showed that PGE2, PGF2 alpha and PGI2 were at the same level as in the non-treated animals, but thromboxane was absent, suggesting that this can be responsible for the progressive dermal ischemia post-electrocution and that the decrease of its synthesis can lead to the increase of the dermal perfusion. 

**Justification of the study **

 Taking into account the incidence and the severity of electrocutions, we consider as extremely necessary to find effective, appropriate and particularized therapeutic solutions aimed at improving the survival, decreasing the mortality, ensuring a superior functional and aesthetic effect and facilitating the social reintegration. 

 The selection of the timing for re-excision is very important, given the severity of the general condition of the electrically injured patient and the fact that any worsening of the lesions has a systemic echo.

 The postponement of the surgical timing can break the precarious metabolic equilibrium and can hasten the installation of multisystem organ failure (MSOF).

**Aim of the study**

 The study is intended to establish a possible connection between the clinical evolution of the electrically injured patient and the dynamics of three important biological parameters, able to provide data concerning the therapeutic attitude to be followed. 

 The patients with diagnostic of high-voltage electrocution, who will be admitted into the Clinic, will be followed for a period of 2 years. The parameters to be followed daily will be: 

 - Creatin-kinase, as a marker of muscular damage 

 - Hemoglobin, as a marker of tissue oxygenation 

 - Leukocytes, as an indicator of a possible septic evolution. 

 The therapeutic alternatives, including the administration of antiplatelet drugs will be studied. 

**Classes of antiplatelet drugs**

 Inhibitors of cyclooxygenase 

 - ADP receptors inhibitors (clopidogrel, prasugrel, ticlopidine) 

 - phosphodiesterase inhibitors (cilostazol)

 - glycoprotein IIb-IIIa inhibitors (abciximab, eptifibatide, tirofiban) 

 - adenosine reuptake inhibitors (dipyridamole) 

 - Inhibitors of thromboxane synthesis 

 - Inhibitors of thromboxane receptors (terutroban) 

 Theoretically, they can study the levels of arachidonic acid decomposition products, thromboxanes and prostaglandins.

 As the determination of thromboxanes level can be made only in research laboratory conditions, we shall rely on the already published studies that connect the thromboxanes to the late ischemia.

 Starting from this information, we want to find out what causal relationship can exist between the administration of antiplatelet agents and the increase of tissue oxygenation or the decrease of the late ischemic effect, evidenced by the more rapid decrease of CK level, the image analysis of the lesions and the Hb level. 

 Following the clinical and paraclinical observations a therapeutic protocol containing the specific medication with anti-ischemic role that can be administered immediately after the arrival of the patient in the specialized service, even in the emergency room, was established. 

 A surgical protocol closely related to the evolution of the biological constants will be established, choosing the optimal timing for re-excision or amputation. 

 In the period October 2010-June 2013 a total of 12 cases of high-voltage electrocution were admitted to our clinic. Among these, 7 cases could be placed in the study, as the remaining patients died within the first 24 hours of hospitalization due to the endured lesions. 

 All the patients were admitted to the ICU ward that supported the treatment and monitoring until their stabilization, at which time they were transferred to the ward. 

 All the patients received anti-thromboxane treatment from their admission (injectable NSAIDs associated with antisecretory drugs). By mutual agreement with ICU service, Dipyridamole was not introduced because of the “steal effect” in the viable areas to the detriment of the already ischemic areas, the drug effect being obvious in vitro, but hard to be proven in the clinical case. 

 The utilization of a treatment with injectable prostaglandins (Ilomedin), successfully used in the arteritis patients and with proven effect of creating neoformation vessels has been taken into discussion. However, taking into account the protocol of utilization extended on several days as well as the unfavorable adverse reactions (decrease of the blood pressure) in an already hemodynamically unstable patient, we gave up using this class of drugs. 

 A trustworthy ally has been the utilization of isogroup blood, isoRh blood as MER or whole blood, as well as of derivatives. We found out that the decrease of Hb under 7 g/dl has an ominous effect on the areas that are uncertain from the tissue oxygenation point of view, a decrease of Hb even for 2-3 hours, being sufficient to determine the extension and the deepening of the ischemic areas. 

 The lesion of electrocution, even more than that of thermal burn should be seen as a constantly evolving lesion, the most common evolution being that to deepening. Our desideratum is to stop the deepening of the lesion, the recruitment of healthy tissue from the contingent of the ischemic one being impossible. An important ally could be the hyperbaric chamber, increasing the amount of oxygen that reaches the tissues in the situation of a poor hematosis. Unfortunately, our service does not have such a device, but the utilization of the hyperbaric chamber could be an integral part of the protocol for the immediate caring of the patient with severe electrocution. 

 The serial excisions are always needed, but never predictable in number. 

 The carrying out of excisions up to the healthy tissue remains a desideratum, in fact being difficult to exactly delineate the viable from non-viable. Even the excision in healthy tissue, without massive hemodynamic support does not have the anticipated result, the lesion deepening again in a few hours. 

 The relationship between the CK level and the clinical appearance of the ischemic areas is relative. We cannot conclude that an increased level of CK is equivalent to an enlarged ischemic area and even less, it does not provide us direct information concerning the best time for re-excision.

 The presence of a viable blood supply around the necrotic tissue will lead to an important resorption of degradation products in that area, a quasi-normal level of CK having no value. The sealing of the necrosis areas and the lack of immediate resorption do not have a positive prognostic value. Taking into account that the electrocutions are mostly multiple injuries, the CK level can increase even after some muscular damage, fractures, independent of the actual electrocution lesion. 

 In one case, the patient suffered from electrocution at both thoracic limbs. With the carbonization of the hands and grifa installed up to the level of the elbow fold, he stayed for 6 hours at the accident site until he was recovered. At the moment of presentation to the hospital, his consciousness condition was satisfactory but the CK level was of over 20000 IU, becoming rapidly non-detectable, in combination with black urine. The patient's condition deteriorated quickly and although a bilateral shoulder disarticulation was carried out, he died in the following 12 hours. 

 As a conclusion, the CK level did not prove itself prognostic for the surgical timing or the actual surgical attitude and could be influenced by a whole series of factors, dependent or not on the electrocution lesion. 

 A radical attitude is to be preferred in cases with established ischemia; the more reserved the prognostic the larger the damage and the longer the period of time from the event. The established treatment is of renal support and treatment of acute renal injury (AKI) subsequently installed. Ultimately, if not per primam, dialysis is performed. From the experience of the cases we had, the increased CK level (over 10000 IU) has a reserved prognostic, translating massive tissue lesions and/or resorption from the ischemic areas with the rapid deterioration of the renal function; the faster it installs the more the electrolyte balance delayed is to be established. 

An increased level of leukocytes is always present as in any severe trauma, even if there are not immediate signs of infection of the electrocution lesions. 

Taking into account that the electrocution lesion as well as the one caused by burning destroys the natural defense barrier represented by the skin, the infection risk is major and that is why the therapeutic protocol stipulates the immediate establishment of a treatment with broad-spectrum antibiotics or with an association of antibiotics. 

 The increase of the leukocytes level under antibiotics treatment involves either the contamination with a germ that is not sensitive to the respective antibiotic or the persistence of necrosis areas, which secondarily infect, and where antibiotic penetration is very low. Therefore, the excision of the compromised tissues is an absolute necessity.

 In terms of prognostic, the increase of the leukocytes number signified an insufficient excision and indicated the resume and deepening of the excisions. Taking into account that the patient has been admitted through the ICU service, the risk of contracting severe infections with selected germs is real. Another risk is that of infection with Clostridium difficile following the prolonged utilization of broad-spectrum antibiotics, especially in patients with associated diseases and reduced immunity per primam. 

 The existence of completely separate circuits should solve the problem of contamination with bacteria of selected species; unfortunately, in our cases, we have faced this problem and the utilization of last choice antibiotics (Imipenem, Vancomycin, Targocid etc.) as well as the association of immunoglobulins was necessary. 

 All the patients admitted in the study received anti-thromboxane drugs in order to limit the ischemic process at tissue level. Despite the efforts we have made, the lack of blood and its derivatives or simply the negligence in patient monitoring, allowed the decrease, even transient of the Hb level, sometimes only for a few hours, but enough to allow the deepening of the ischemic lesions. 

 In all the patients, excisions in emergency or even amputations of the extremities were carried out, with the desire to limit the extension of the ischemic lesions and the resorption of cell degradation products.

 The amputations performed in emergency did not always represent a saving solution; however, they remained the most effective measures when they were carried out immediately after the accident and obviously in viable tissue. 

 The increase of CK is not an indicative factor in making re-excisions itself, but orients the therapeutic approach, the utilization of the dialysis being required when the values do not decrease by treatment for renal support and forcing of diuresis. 

 The normalization of CK indicates the time when we can start the covering of the defects resulted because of the excisions.

 The level of the leukocytes represents both a prognostic factor and an indicative factor for the re-excision of the ischemic areas. An increased level under antibiotic therapy signifies either an incomplete excision or the contamination with flora resisting to the antibiotic that has been used. 

 In the light of findings in the caring of the patients with electrocutions, I propose several caring/assessment protocols for the severe electrically injured patient. 

 The evaluation/monitoring protocol should contain the level of Hb and tissue O2, which are the main constants that vary at the time of installation and deepening of the ischemic lesions appearing in electrocution. The decrease of the Hb under 7 g/dl is translated by the deepening and expanding of the ischemic lesions. 

 CK monitoring has a prognostic role, being useful in modulating the renal support therapy, indicating the setting up of treatment by dialysis and establishing the moment when the covering of the tissue defects can be started. 

 The monitoring of the leukocyte level establishes the indication of re-excision, of setting up/change of the antibiotics treatment, together with CK indicating the beginning of the surgical procedures for tissue covering.

 The surgical protocol involves the performance in emergency conditions of excisions of the ischemic lesions, necessity amputations, and incisions for decompression.

 The excisions are always serial, with a great difficulty in the separation of the healthy tissue from the ischemic one, the evolution in the dynamics of lesions being of the order of hours. 

 Sometimes, an emergency reconstructive procedure may be required, as it is for the lesions of denuded magistral vessels, because of the serial excisions, knowing that the vessel is protected from the surrounding muscular mass by the blood flow, which decreases the local temperature.

 The procedures for the covering of the tissue defects can be simple or complex depending on the case peculiarity from direct sutures to skin transplants, pedicle flaps, free tissue transfer flaps, etc. It is always a long-lasting process that takes place in several stages.

 For the limitation of the tissue ischemia, for the contraction of the extended and atone wounds we have also used, with good results, some conservative methods like insulin and Platelet-Rich Plasma (PRP) injections. 
